# 

**Published:** 1886-04

**Authors:** 


### ANNOUNCEMENTS.

THE AMERICAN MEDICAL ASSOCIATION.—The thirty-seventh annual session will be held in St. Louis, Mo., on Tuesday, Wednesday, Thursday and Friday, May 4, 5, 6, and 7, commencing on Tuesday, at 11 A. M.

"The delegates shall receive their appointment from permanently organized State Medical Societies, and such County and District Medical Societies as are recognized by representation in their respective State Societies, and from the Medical Department of the Army and Navy and the Marine Hospital Service of the United States.

"Each State, County, and District Medical Society entitled to representation shall have the privilege of sending to the Association one delegate for every ten of its regular resident members, and one for every additional fraction of more than half that number: *Provided*, however, that the number of delegates for any particular state, territory, county, city, or town shall not exceed the ratio of one in ten of the resident physicians who may have signed the code of ethics of the Association."

*Sections.*—"The Chairman of the several Sections shall prepare and read, in the general sessions of the Association, papers on the advances and discoveries of the past year in the branches of science included in their respective Sections. * * * *."—*By-Laws, Art. II, Sec. 4.*

Practice of Medicine, Materia Medica, and Physiology.—DR. J. T. WHITTAKER, Cincinnati, Ohio, Chairman; DR. B. L. COLEMAN, Lexington, Ky., Secretary.

Obstetrics and Diseases of Women and Children.—DR. S. C. GORDON, Portland, Me., Chairman; DR. J. F. Y. PAINE, Galveston, Texas, Secretary.

Surgery and Anatomy.—DR. NICHOLAS SENN, Milwaukee, Wis., Chairman; DR. H. H. MUDD, St. Louis, Mo., Secretary.

State Medicine.—DR. JOHN H. RAUCH, Springfield, Ill., Chairman; DR. F. E. DANIEL, Austin, Texas, Secretary.

Ophthalmology, Otology, and Laryngology.—DR. EUGENE SMITH, Detroit, Mich., Chairman; DR. J. F. FULTON, St. Paul, Minn., Secretary.

Diseases of Children.—DR. W. D. HAGGARD, Nashville, Tenn., Chairman; DR. W. B. LAWRENCE, Batesville, Ark., Secretary.

Oral and Dental Surgery.—DR. JOHN S. MARSHALL, Chicago, Ill., Chairman; DR. A. E. BALDWIN, Chicago, Ill., Secretary.

A member desiring to read a paper before a Section should forward the paper, or its title and length (not to exceed twenty minutes in reading), to the Chairman of the Committee of Arrangements, at least one month before the meeting.—*By-Laws.*

Committee of Arrangements.—DR. LE GRAND ATWOOD, St. Louis, Mo., Chairman.

THE AMERICAN SURGICAL ASSOCIATION.—The next annual meeting of the American Surgical Association will be held in Washington, D. C., on April 28th, 29th, 30, and May 1st.

THE ILLINOIS STATE MEDICAL SUCIETY.—The Thirty-Sixth Annnal Meeting of the Illinois State Medical Society will be held in Bloomington ,beginning on the third Teusday in May, at 10 A. M.

### BOOKS RECEIVED.

*The Methods of Bacteriological Investigation.* By Dr. Ferdinand Hueppe. New York: D. Appleton & Co. Chicago: Jansen, McClurg & Co.

*How We Treat Wounds To-day.* By Robert T. Morris, M.D. New York: G. P. Putnam's Sons. Chicago: W. T. Keener.

*The Diagnosis and Treatment of Diseases of the Ear.* By Oren D. Pomeroy, M.D. New York: D. Appleton & Co. Chicago: Jansen, McClurg & Co.

*A Manual of Auscultation and Percussion.* By Austin Flint, M.D., LL.D. Philadelphia: Lea Brothers & Co. Chicago: W. T. Keener.

*Fractures and Dislocations.* By T. Pickering Pick, F.R.C.S. Philadelphia: Lea Brothers & Co. Chicago: Jansen, McClurg & Co.

*Seventh Annual Report of the State Board of Health of Illinois.*

*Practical Human Anatomy.* By Faneuil D. Weisse, M.D. New York: William Wood & Co. Chicago: W. T. Keener.

*Essentials of the Physical Diagnosis.* By E. Darwin Hudson, Jr., A.M., M.D. New York: Styles & Cash.

*Tablets of Anatomy.* By Thomas Cooke, F.R.C.S. London: Longmans Green & Co.

*A Treatise on the Diseases of Infancy and Childhood.* By J. Lewis Smith, M.D. Philadelphia: Lea Brothers & Co. Chicago: Jansen, McClurg & Co.

*Local Anæsthesia in General Medicine and Surgery.* By J. Leonard Corning, M.D. New York: D. Appleton & Co. Chicago: Jansen, McClurg & Co.

*The Field and Limitation of Operative Surgery of the Human Brain.* By John B. Roberts, A.M., M.D. Philadelphia: P. Blakiston Son & Co. Chicago: W. T. Keener.

*A Guide to the Practical Examination of the Urine.* By James Tyson, M.D. Philadelphia: P. Blakiston Son & Co. Chicago: W. T Keener.

*Handbook of the Diseases of the Nervous System.* By James Ross, M.D., LL.D. Philadelphia: Lea Brothers & Co. Chicago: Jansen, McClurg & Co.

### PAMPHLETS RECEIVED.

*A Case of Prolonged Gestation with Autopsy of the Fœtus.* By M. Nunez Rossie, M.D. Manuel de Technique des Autopsies. Par Bourneville and P. Bricon.

*What is Medicine?* By Albert L. Gihon, A.M., M.D.

*Transactions of the Medical Society of the State of Pennsylvania.*

*Eczema.* By George H. Rohe, M.D.

*Progress of Electrolysis in Surgery.* By Robert Newman, M. D.

*Insidious Septicæmia—A Rare, Deceptive, and Fatal Form of the Disease* By George J. Engelmann, M.D.

*A New Departure in Uterine Therapeutics.* By George J. Engelmann, M.D.

*Puerperal Pyrexia.* By George P. Andrews, M.D.

*First Annual Report of the Pathological Department Norristown State Hospital.*

*The Causation and Nature of Hypertrophy of the Prostate.* By Reginald Harrison, F.R.C.S.

*Cocaine in Hay-Fever.* By Seth S. Bishop, M.D.

*Proceedings and Addresses at the Sanitary Convention, held in Ypsilanti, Michigan, June 30th and July 1st, 1885.*

*Twelfth Annual Report of the Superintendent of the Cincinnati Sanitarium.*

*Report of an Inspection of the Atlantic and Gulf Quarantines.* By John H. Rauch, M.D.

3cm
LIBRARY INDEXED S.G.O.

SUBSCRIPTION PRICE, $3.00 PER YEAR.

Whole Number, 316. APRIL, 1886. Vol. LII., Number 4

THE

### CHICAGO MEDICAL JOURNAL AND EXAMINER

EDITORS:

S. J. JONES, M.D., LL.D.

W. W. JAGGARD, A.M, M.D. HAROLD N. MOYER, M.D.

COLLABORATORS.

CHICAGO:

PROFESSOR J. A. ALLEN, M. D., LL. D.
PROFESSOR E. ANDREWS, M. D., LL. D.
PROFESSOR W. H. BYFORD, A. M., M. D.
PROFESSOR O. C. DEWOLF, A. M., M. D.
PROFESSOR E. C. DUDLEY, A. B., M. D.
PROFESSOR J. H. ETHERIDGE, A. M., M. D.
PROFESSOR C. FENGER, M. D.
PROFESSOR J. N. HYDE, A. M., M. D
PROFESSOR E. F. INGALS, A. M., M.D.
PROFESSOR H. A. JOHNSON, M. D., LL. D.
CHARLES GILMAN SMITH, A. M., M.D,
J. F. TODD, M. D.

MILWAUKEE, WISCONSIN:
PROFESSOR N. SENN, M. D.

WASHINGTON, D. C:
S. O. RICHEY, M. D.

UNITED STATES ARMY:
SURGEON, D. L. HUNTINGTON, M. D.

UNITED STATES NAVY:
MEDICAL DIRECTOR, J. M. BROWNE, M. D
MEDICAL DIRECTOR, A. L. GIHON, A. M., M. D.

